# Yaws

**DOI:** 10.1093/bmb/ldu037

**Published:** 2014-12-18

**Authors:** Michael Marks, Oriol Mitjà, Anthony W. Solomon, Kingsley B. Asiedu, David C. Mabey

**Affiliations:** † Clinical Research Department, Faculty of Infectious and Tropical Diseases, London School of Hygiene and Tropical Medicine, Keppel Street, London WC1E 7HT, UK; ‡ Hospital for Tropical Diseases, University College London Hospitals NHS Trust, Mortimer Market, London WC1E 6JB, UK; § Barcelona Centre for International Health Research,Hospital Clinic, University of Barcelona, Roselló 132, Barcelona, Spain; ** Lihir Medical Centre-International SOS, Newcrest Mining, Lihir Island, Papua New Guinea; †† Department of Control of Neglected Tropical Diseases, World Health Organization, Avenue Appia 20, 1211 Geneva 27, Switzerland

**Keywords:** yaws, syphilis, eradication, neglected tropical diseases

## Abstract

**Introduction:**

Yaws, caused by *Treponema pallidum* ssp. *pertenue*, is endemic in parts of West Africa, Southeast Asia and the Pacific. The WHO has launched a campaign based on mass treatment with azithromycin, to eradicate yaws by 2020.

**Sources of data:**

We reviewed published data, surveillance data and data presented at yaws eradication meetings.

**Areas of agreement:**

Azithromycin is now the preferred agent for treating yaws. Point-of-care tests have demonstrated their value in yaws.

**Areas of controversy:**

There is limited data from 76 countries, which previously reported yaws. Different doses of azithromycin are used in community mass treatment for yaws and trachoma.

**Growing points:**

Yaws eradication appears an achievable goal. The programme will require considerable support from partners across health and development sectors.

**Areas timely for developing research:**

Studies to complete baseline mapping, integrate diagnostic tests into surveillance and assess the impact of community mass treatment with azithromycin are ongoing.

## Introduction

Yaws is an infectious disease caused by *Treponema pallidum* ssp. *pertenue* and is one of the four treponemal diseases affecting humans.^1,2^ Unlike syphilis, which is caused by the almost identical *T. pallidum pallidum*, it is not sexually transmitted, but is thought to be spread by skin to skin contact in warm humid environments, and mother to child transmission is not seen. As with other treponemal infections, yaws causes primary, secondary and tertiary lesions, which predominantly affect the skin, bones and cartilage. Yaws was the first disease to be targeted for eradication by the World Health Organization (WHO), and mass screening and treatment programmes led by WHO reduced the global prevalence by >95% between 1950 and 1964, but it has re-emerged as an important public health problem in West Africa, Southeast Asia and the Pacific in recent years.^3^ The recent demonstration that a single oral dose of azithromycin is as effective as injectable penicillin in the treatment of yaws^4^ has prompted renewed interest in the possibility of yaws eradication, but barriers such as funding and access to azithromycin have to be overcome before this goal can be realized.

## Epidemiology

Yaws is found in warm and humid environments^5^ and affects mostly children between 2 and 15 years old, who are considered as the reservoir for infections. The disease is spread by direct skin-to-skin, non-sexual, contact often after a cut or abrasion in the lower legs.^6^ There have been suggestions that flies may act as a vector for yaws,^6^ but there is no definitive proof that this occurs. Treponemal infections closely related to yaws and syphilis have been identified in primates, but there is no evidence to suggest that zoonotic transmission between humans and non-human primates occurs.^7^ Children born to mothers affected with yaws are generally unaffected, and most evidence seems to indicate that the disease is not acquired congenitally.

The early lesions of yaws are most infectious. It is estimated that patients are infectious for up to 12–18 months following primary infection,^6^ but relapsing disease can extend this period. The destructive lesions of late yaws are not infectious. In studies in both Papua New Guinea and the Solomon Islands, endemicity at the village level has been identified as the major risk factor for infection and re-infection following treatment.^8,9^ The disease primarily affects rural communities with low standards of hygiene, with incidence declining as social and economic status rise.

In the mid-20th century, yaws was reported to affect ∼50 million individuals and to be endemic in at least 90 countries^10^ in South America, the Caribbean, Africa, Asia and the Pacific. The WHO launched a major eradication effort in the 1950s based on mass screening and treatment with injectable penicillin. The campaign examined some 300 million individuals of whom 50 million were treated. Although yaws was not eradicated, by the end of the major campaign in 1964, the burden of yaws had been significantly reduced to ∼2.5 million cases.^3^

Following this initial success of the WHO campaign, yaws dropped down the public health agenda internationally and domestically in many countries. In the 1970s and 1980s, there was a resurgence of cases in some countries in West and Central Africa.^11,12^ This led to a renewal of control efforts, which again reduced the burden of the disease but did not eradicate it.

**Fig. 1:**
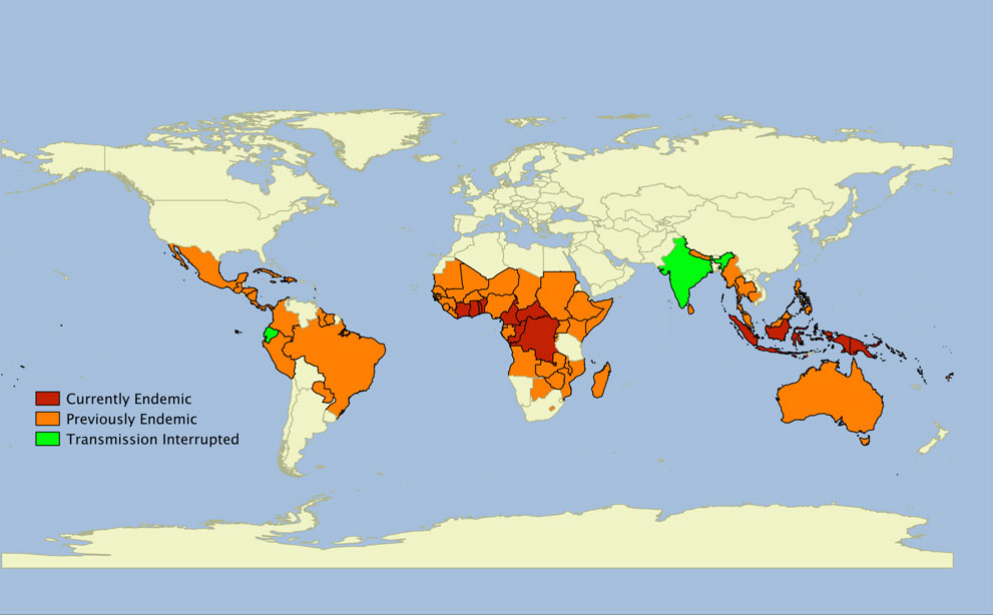
Worldwide distribution of yaws. Data taken from the World Health Organization. Global Health Observatory Data Repository. http://apps.who.int/gho/data/node.main.NTDYAWSEND?lang=en (2 October 2014, date last accessed).

Over the past 20 years, there has been a further resurgence of yaws in previously endemic countries, and the disease is now thought to be endemic in at least 12 countries in West Africa, Southeast Asia and the Pacific. There are a further 76 countries that previously reported yaws, throughout Africa, the Americas, Asia and the Pacific, for which adequate up-to-date surveillance data are not currently available (Fig. 1).^10^ Most yaws cases are concentrated in just three countries: Ghana, Papua New Guinea and the Solomon Islands have each reported >15 000 cases annually within the last 3 years. In another eight countries, transmission occurs in focal communities. Despite being deprioritized in international health fora, both India and Ecuador have reported eliminating yaws in recent years with prolonged campaigns based on case identification, contact tracing and treatment with injectable penicillin,^13^ demonstrating that sustained efforts can be successful.

## Bacteriology

*Treponema pallidum* is a spirochaete that cannot be cultured *in vitro*.^6^ They divide slowly (every 30 h), have a characteristic corkscrew-like motility and can move through gel-like environments such as connective tissue. They are rapidly killed by drying, oxygen exposure or heating, and they cannot survive outside the mammalian host. The four pathogenic treponemes are morphologically and serologically indistinguishable, and share at least 99% DNA sequence homology.^14^ Whole-genome sequencing has demonstrated that the genome of *T. p* ssp. *pertenue* differs by only 0.2% from that of *T. p* ssp. *pallidum*,^14^ the causative organism of venereal syphilis. The phylogenetic relationship between different subspecies of treponemes is not clear, as very few isolates of the non-venereal subspecies are available.^15^

## Clinical features

As with other treponemal diseases, the clinical features of yaws may be conveniently divided into primary, secondary and tertiary disease.^2,6,16^ Although this classification is clinically useful, it should be remembered that patients may present with a mixture of clinical signs.

### Primary yaws

**Fig. 2:**
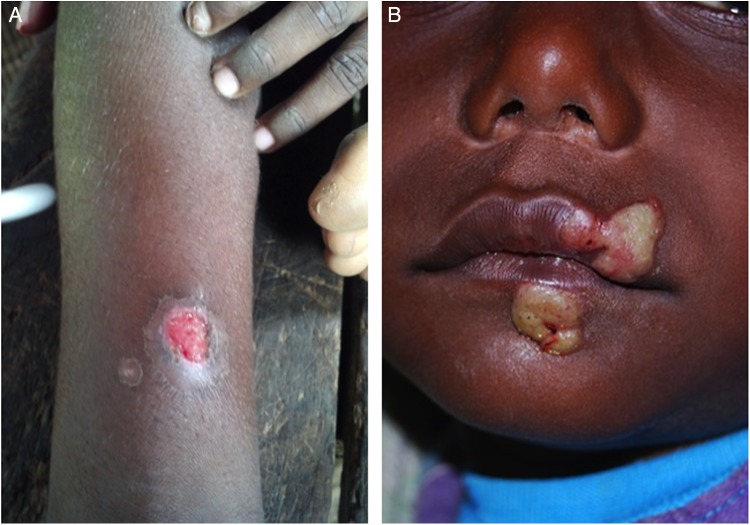
Lesions of primary yaws. **A**, typical ulcer of primary yaws. **B**, papilloma of primary yaws (Images reproduced with permission of M.M. and O.M.).

The initial lesion of primary yaws is a papule appearing at the site of inoculation after ∼21 days (range 9–90 days).^6^ This ‘Mother Yaw’ may evolve either into an exudative papilloma, 2–5 cm in size, or degenerate to form a single, crusted, non-tender ulcer (Fig. 2). The lower limbs are the commonest site for primary yaws lesions, but other parts of the body may all be affected. Unlike venereal syphilis, genital lesions are extremely uncommon. In untreated individuals, primary lesions may heal spontaneously over a period of 3–6 months, leaving a pigmented scar.^17^ Primary lesions may still be present in patients who present with secondary yaws.

### Secondary yaws

After a period of 1–2 months (sometimes up to 24 months), haematogenous and lymphatic spread of treponemes may result in progression to secondary yaws, which predominantly affects the skin and bones,^18,19^ often with general malaise and lymphadenopathy.

**Fig. 3:**
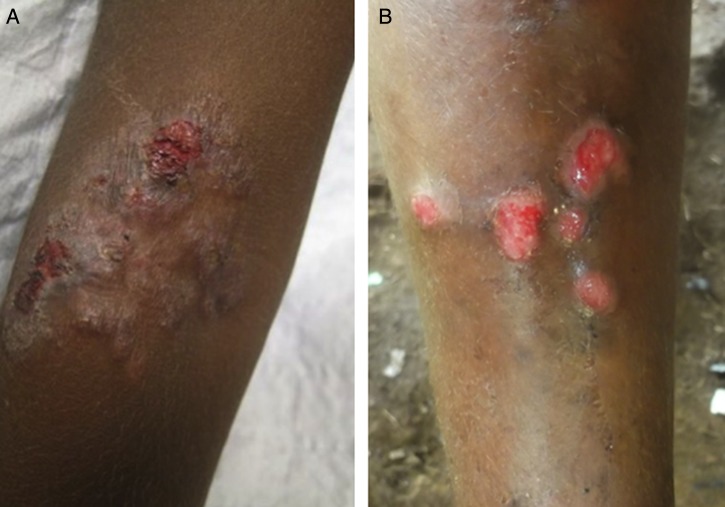
Skin lesions of secondary yaws. **A**, Crusted maculopapular lesion of secondary yaws. **B**, Multiple ulcers of secondary yaws (Images reproduced with permission of M.M. and O.M.).

As with venereal syphilis, a wide range of skin manifestations has been described in secondary yaws (Fig. 3). Patients may develop disseminated papillomatous or ulcerative lesions, scaly macular lesions or hyperkeratotic lesions on the palms and soles.^6,19^ Hyperkeratotic lesions can crack and become secondarily infected, resulting in severe pain and a crab-like gait (crab-yaws).^20^ Mucous membrane involvement is uncommon in secondary yaws.^21^

**Fig. 4:**
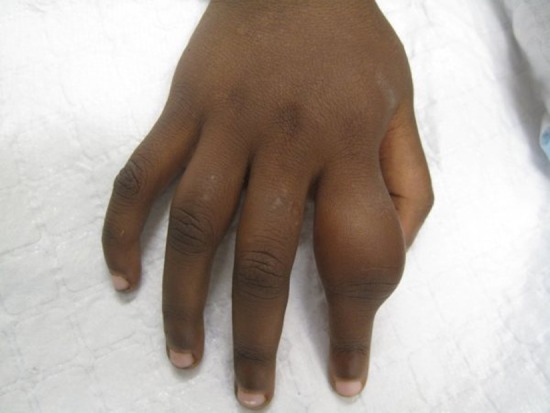
Bony lesions of secondary yaws. Dactylitis due to secondary yaws (Images reproduced with permission of O.M.).

Alongside the skin, involvement of the bones is one of the cardinal features of secondary yaws. The most common manifestation is osteoperiostitis, involving the fingers (resulting in dactylitis) or long bones (forearm, fibula and tibia) which results in bony swelling and pain (Fig. 4).^18^ In most patients, multiple bones can be affected. In a study from Papua New Guinea,^19^ 75% of children with secondary yaws had joint pain. Following treatment of primary or secondary yaws, skin lesions usually resolve within 2–4 weeks and bone pain may begin to resolve in as little as 48 h.^1,2^

As in all treponemal infections, untreated patients may develop latent infection, with positive serology but no clinical signs. Latent cases can relapse, usually in the first 5 years (rarely up to 10 years) after infection.^6^ Relapsing lesions tend to occur around the axillae, anus and mouth.

### Tertiary yaws

The destructive lesions of tertiary yaws were previously reported to occur in up to 10% of untreated patients but are now rarely seen. As in other stages of the disease, the skin is most commonly affected. Nodular lesions may occur near joints and ulcerate, causing tissue necrosis.^5^ Destructive lesions of the face were one of the most marked manifestations of late-stage yaws. *Gangosa*, a destructive osteitis of the palate and nasopharynx, results in mutilating facial ulceration. *Goundou*, which was rarely reported even when yaws was hyperendemic, is characterized by exostoses of the maxillary bones.^2^

Unlike syphilis, yaws is not thought to cause cardiovascular or neurological disease.^2^ Post-mortem studies in a yaws-endemic community in Ghana found that aortitis, histologically similar to that found in tertiary syphilis, was the most common cardiovascular abnormality, but definitive evidence that this was due to yaws is lacking.

## Attenuated disease

The manifestations of yaws appear to be less florid than previously described.^22^ In particular, tertiary manifestations are now rarely seen. There is no agreed definition of attenuated yaws, nor a clear explanation for why the clinical features of the disease may have changed, although improvements in living standards, use of treponemocidal antibiotics for other infections and mutations in *T. p* ssp. *pertenue* have all been proposed.^22^

### Diagnosis

*Treponema pallidum* is not viable *ex vivo*, which has limited the value of direct diagnostic methods. While dark field microscopy allows direct visualization of spirochaetes,^6^ the skills and equipment required are not available in most locations even in relatively high-income settings. Instead diagnosis has rested on combinations of serological assays and, more recently, nucleic acid amplification tests (NAATs).

As with venereal syphilis, serology has been the mainstay of laboratory diagnosis.^23^ Serological detection of yaws requires detection of two distinct antibodies: one against a treponemal antigen and one against a non-treponemal antigen. *Treponema pallidum* particle agglutination (TPPA) and haemagglutination (TPHA) assays are used to detect *Treponema*-specific antibodies. Once positive, these tests usually remain positive for life. The venereal disease research laboratory (VDRL) and rapid plasma reagin (RPR) tests are non-treponemal tests, which may give rise to biological false positives, but more accurately reflect active disease and can be used as a test of cure, since titres fall following successful treatment.

A major challenge for clinicians and epidemiologists has been that the pathogenic treponemes are serologically indistinguishable. Given the considerable epidemiological and clinical overlap between the syndromes, this continues to represent a barrier to accurate data on the incidence and prevalence of yaws.

While serological assays do not require sophisticated equipment, they do require access to laboratory facilities, which are rarely available to the remote communities where yaws is endemic. Rapid diagnostic tests (RDTs) have proved effective in the diagnosis of syphilis,^24^ and a number of evaluations of their performance in diagnosing yaws have recently been undertaken. In Papua New Guinea, an RDT (Chembio Diagnostic System, Inc., New York, NY, USA) that detects both treponemal and non-treponemal antibodies was shown to be valuable in providing serological confirmation of clinically suspected cases.^25^ In the Solomon Islands, the same RDT was shown to be of use for community surveillance and mapping.^26^ These tests may help to improve reporting practices for yaws worldwide.

Polymerase chain reaction (PCR) tests have been commonly adopted for the diagnosis of venereal syphilis, but distinguishing between subspecies of *T. pallidum* currently relies on combined PCR and sequencing^27^ and is only available at research laboratories. PCR has also emerged as a tool for diagnosing other causes of skin lesions in yaws-endemic populations, including *Haemophilus ducreyi*.^28,29^ Wider access to NAAT-based diagnostics, including possible point-of-care tests, will be required as part of the WHO yaws eradication programme.

### Diagnostic quandaries

The differential diagnosis of tropical ulcerative lesions is broad^30^ and varies depending on the stage and type of lesion. As examples, the primary lesions of yaws may be mistaken for cutaneous leishmaniasis, tropical ulcer caused by fusobacteria and Treponema vincentii, or pyoderma. Of particular importance is the emergence of *H. ducreyi*, the causative organism of the sexually transmitted disease chancroid, as a common cause of non-genital skin lesions in a number of countries where yaws is endemic. There are now reports of *H. ducreyi* as a cause of non-genital skin lesions from Papua New Guinea, the Solomon Islands, Ghana and Vanuatu.^28,29^ Data from experimental models of chancroid suggest that these lesions should be responsive to azithromycin,^31^ and therefore, that mass distribution of azithromycin for yaws (and possibly trachoma) could also be effective in treating lesions resulting from infection with *H. ducreyi*.

### Treatment

Long acting, injectable penicillin has been the mainstay of treatment for yaws for over 50 years^6^ and was the cornerstone of all previous yaws control and eradication programmes. Despite extensive use, *T. pallidum* remains exquisitely sensitive to penicillin, with no evidence that resistance has emerged. There have been rare reports of treatment failure following treatment with penicillin, but the difficulty of distinguishing treatment failure from reinfection makes the significance of these findings uncertain.^32^ The major drawback of injectable penicillin has been the requirement for trained medical staff to administer treatment, the risk of transmitting blood-borne infections and the possible risk of anaphylaxis. The recommended doses of benzathine penicillin for the treatment of yaws (1.2 MU for adults, 0.6 MU for children) are lower than those used in venereal syphilis,^6^ and in situations where the diagnosis is unclear, clinicians are advised to treat for syphilis.^16^

Before 2012, no other agents had been evaluated against yaws in randomized controlled trials, but there are published observational data suggesting prolonged courses of oral penicillin or tetracyclines could be effective.^33,34^ Erythromycin has also been recommended based on its efficacy in the treatment of venereal syphilis. These treatment options are of less relevance since the emergence of azithromycin as an effective treatment for yaws.

The macrolide antibiotic azithromycin was previously shown to be effective in treating venereal syphilis.^35^ A landmark paper published in 2012 compared a single oral dose of azithromycin (30 mg/kg) to benzathine penicillin in the treatment of primary and secondary yaws.^4^ Azithromycin was non-inferior to penicillin, with clinical and serological cure in 96% of individuals randomized to treatment with azithromycin.

Azithromycin has a number of advantages as an agent in the treatment of yaws. It is orally administered and has a favourable safety profile. It has been widely and successfully used in mass drug administration programmes for the control and elimination of trachoma.^36^ One area of concern is the possibility of resistance to azithromycin, which is now widespread in sexually transmitted strains of *T. pallidum*.^37,38^ Monitoring for the development of resistance in *T. p* ssp. *pertenue* will be an extremely important component of the WHO yaws eradication strategy.

## Eradication efforts

The emergence of azithromycin as an effective, single-dose oral agent for the treatment of yaws has led to renewed interest in the disease. In 2012, the WHO outlined a new strategy (the Morges strategy) for yaws eradication.^39^ This strategy is based on community mass treatment with single-dose oral azithromycin, with subsequent clinical case detection to direct further rounds of mass or targeted treatment with azithromycin. The WHO is aiming to eradicate the disease by 2020.^39^

The previous WHO programmes in the 1950s to 1960s resulted in significant reductions in the worldwide burden of yaws^3^ but did not successfully eradicate it. It is thought that a failure to adequately identify and treat contacts and latent cases alongside a lack of integration of yaws surveillance in to national health programmes were responsible for thisfailure.^40^

Both India and Ecuador have reported eliminating yaws since 2000, and their experiences are informative for the current global eradication campaign. Ecuador experienced a large drop in yaws incidence and prevalence following the initial WHO campaigns of the 1950s,^13^ but further control efforts were complicated by the anecdotal nature of case reporting. Ecuador instituted a more sustained surveillance programme in the late 1980s, combining continuous village-level monitoring for skin lesions with formal surveys conducted every 5 years that included clinical and serological screening. Individuals identified as having yaws (active or latent) were treated with injectable benzathine penicillin. In surveys conducted between 1988 and 1993, the prevalence of yaws dropped by over 90%, and in a follow-up survey conducted in 1998, no new cases were been detected.^13^

In India, initial eradication efforts were launched in the 1950s, but the disease rebounded in the 1970 and 1980's. In 1996, the government launched a yaws eradication programme.^41^ As in Ecuador, the programme involved a combination of clinical screening and treatment with intermittent serological surveys. A notable aspect was the provision of financial incentives for individuals to report suspected cases to the control programme.^41^ As in Ecuador, the programme drove a sustained reduction in yaws incidence from 3571 cases in 1996 to 0 cases in 2004.

Despite optimism surrounding mass distribution of azithromycin, there remain major barriers to a successful eradication programme. Notably, there are limited accurate epidemiological data from many countries where yaws is currently reported. Most national surveillance systems report clinically suspected cases only, without serological or PCR confirmation. Given the wide range of phenotypically similar skin lesions that may occur, it is likely that these figures are inaccurate. Capacity building to support improved surveillance is a central component of the current eradication campaign. This is compounded by the absence of recent data from many countries where yaws was previously reported to be endemic.^10^ Significant investment in mapping and epidemiology will be required to go along with the eradication programme. Given the ambitious timescales involved, innovative approaches combining mapping with other NTD or disease mapping activities should be considered.

The eradication programme also lacks dedicated funding and a drug donation programme. The cost of eradication has been estimated to be in the region of $112 million dollars, excluding drug costs, with a cost of ∼$21 per DALY saved^42^ that compares favourably to many other public health interventions. As data from a larger number of previously endemic countries become available, it is possible that the total cost of the eradication programme may be revised upwards substantially, although efficiencies of scale may bring the cost per DALY down.

Yaws is known to be endemic in several countries where trachoma is also found, including the Solomon Islands and Vanuatu, and co-ordinated mapping efforts have been undertaken or proposed in some of these countries. As both diseases are controlled by mass distribution of azithromycin, and there is already a dedicated drug donation scheme for trachoma elimination, the possibility of integrating yaws and trachoma control efforts warrants further study. Particular attention will need to be paid to the efficacy of the lower dose of azithromycin (20 mg/kg) used in trachoma control programmes for the treatment of yaws.

## Conclusions

Yaws remains a problem for poor, rural communities in many countries and places a significant burden on national health-care systems. As with the other treponemal diseases, it has a multi-stage disease course predominantly involving the skin and bones. Yaws remains sensitive to penicillin, but azithromycin has emerged as an effective treatment that will be the cornerstone of the WHO-led eradication efforts, providing that it can be delivered to the populations that need it.

The eradication of yaws will require considerable support from partners across the health and development sectors, and a number of challenges need to be overcome for this effort to be successful. Accurate epidemiological data are lacking from both currently and formerly endemic countries, and a significant investment to improve this situation is urgently needed. The development and validation of near-patient and laboratory tests specific for treponemal subspecies are urgently required, in view of the increasing recognition that other bacteria can cause phenotypically indistinguishable skin lesions. Integration of these tools, and monitoring for the emergence of macrolide resistance, will be of critical importance as the programme moves forward. Despite these concerns, significant progress has been made in the last 5 years, raising hopes that yaws eradication may finally become possible.

## Authors’ contributions

M.M. wrote the first draft of the manuscript and made subsequent revisions. O.M., A.W.S., K.B.A. and D.C.M. reviewed and edited the manuscript. M.M. is the guarantor of the paper. K.B.A. and A.W.S. are staff members of the World Health Organization. The author alone is responsible for the views expressed in this article and they do not necessarily represent the decisions or policies of the World Health Organization.

## Bibliographic information

M.M. is a research fellow at the London School of Hygiene and Tropical Medicine. He works on the epidemiology of yaws in the Solomon Islands. O.M. is an infectious disease physician working in Papua New Guinea on the epidemiology, treatment and control of Yaws. A.W.S. is the WHO Medical Officer for trachoma and involved in co-ordinating mapping of yaws and trachoma in co-endemic countries. K.B.A. is the WHO Medical Officer for yaws. D.C.M. is professor of communicable diseases at the London School of Hygiene and Tropical Medicine and a world expert on treponemal infections.

## Funding

M.M. is supported by a Wellcome Trust Clinical Research Fellowship—102807. Funding to pay the Open Access publication charges for this article was provided by the Wellcome Trust.

## Conflict of interest

The authors have no potential conflicts of interest.
